# Investigating dynamic brain functional redundancy as a mechanism of cognitive reserve

**DOI:** 10.3389/fnagi.2025.1535657

**Published:** 2025-02-04

**Authors:** Julia Schwarz, Franziska Zistler, Adriana Usheva, Anika Fix, Sebastian Zinn, Juliana Zimmermann, Franziska Knolle, Gerhard Schneider, Rachel Nuttall

**Affiliations:** ^1^Department of Anesthesiology and Intensive Care, School of Medicine and Health, Technical University of Munich, Munich, Germany; ^2^Department of Anesthesiology, Columbia University, New York, NY, United States; ^3^Department of Neuroradiology, School of Medicine and Health, Technical University of Munich, Munich, Germany; ^4^TUM-Neuroimaging Center, School of Medicine and Health, Technical University of Munich, Munich, Germany

**Keywords:** cognitive reserve, brain functional redundancy, age-related brain changes, functional magnetic resonance imaging (fMRI), dynamic functional connectivity

## Abstract

**Introduction:**

Individuals with higher cognitive reserve (CR) are thought to be more resilient to the effects of age-related brain changes on cognitive performance. A potential mechanism of CR is redundancy in brain network functional connectivity (BFR), which refers to the amount of time the brain spends in a redundant state, indicating the presence of multiple independent pathways between brain regions. These can serve as back-up information processing routes, providing resiliency in the presence of stress or disease. In this study we aimed to investigate whether BFR modulates the association between age-related brain changes and cognitive performance across a broad range of cognitive domains.

**Methods:**

An open-access neuroimaging and behavioral dataset (*n* = 301 healthy participants, 18–89 years) was analyzed. Cortical gray matter (GM) volume, cortical thickness and brain age, extracted from structural T1 images, served as our measures of life-course related brain changes (BC). Cognitive scores were extracted from principal component analysis performed on 13 cognitive tests across multiple cognitive domains. Multivariate linear regression tested the modulating effect of BFR on the relationship between age-related brain changes and cognitive performance.

**Results:**

PCA revealed three cognitive test components related to episodic, semantic and executive functioning. Increased BFR predicted reduced performance in episodic functioning when considering cortical thickness and GM volume as measures of BC. BFR significantly modulated the relationship between cortical thickness and episodic functioning. We found neither a predictive nor modulating effect of BFR on semantic or executive performance, nor a significant effect when defining BC via brain age.

**Discussion:**

Our results suggest that BFR could serve as a metric of CR when considering certain cognitive domains, specifically episodic functioning, and defined dimensions of BC. These findings potentially indicate the presence of multiple underlying mechanisms of CR.

## 1 Introduction

The concept of “reserve” arose from findings of a discrepancy between brain pathology and clinical symptomology (Crystal et al., 1988; Katzman et al., 1988; Morris et al., 1996; Price and Morris, 1999; Mortimer et al., 2003) leading to the suggestion of a certain factor that modulates the relationship between pathological or age-related brain changes and clinical symptom severity or cognitive performance. The cognitive reserve hypothesis—similarly to the Scaffolding Theory of Aging and Cognition (STAC) (Reuter-Lorenz and Park, 2014)—posits that people with a higher cognitive reserve, which is accumulated across the lifespan (Richards and Deary, 2005), can withstand a greater burden of these brain changes whilst showing similar clinical symptom severity or cognitive performance as compared to those with a lower burden and a lower cognitive reserve (Stern, 2002; Steffener and Stern, 2012). Cognitive reserve, although frequently measured through proxies such as educational and occupational attainment (Stern et al., 1994; Richards and Sacker, 2003; Bennett et al., 2003; Staff et al., 2004; Valenzuela and Sachdev, 2006), has been suggested to originate from certain characteristics of the functional networks of the brain that are linked to task performance, such as efficiency, flexibility and compensatory ability (Stern et al., 2005; Stern, 2009; Reuter-Lorenz and Park, 2014; Cabeza et al., 2018; Stern et al., 2019). This is supported by cross-sectional studies involving performance of certain demanding cognitive tasks across young and old participants (Zarahn et al., 2007; Holtzer et al., 2009).

Recent studies have sought to elucidate the mechanisms of cognitive reserve (Stern et al., 2007; Steffener et al., 2011; Speer and Soldan, 2014; Franzmeier et al., 2017; Solé-Padullés et al., 2009; Anthony and Lin, 2017), applying graph theory metrics to uncover various organizational properties of functional brain networks as possible sources of cognitive reserve (Varangis et al., 2019; Sadiq et al., 2021). “Redundancy” in brain network functional connectivity describes the presence of multiple independent pathways between brain regions that serve as “back-up” information processing routes, creating a protective structure which is resistant to cognitive decline as a result of pathological or age-related brain changes. This implies that with a higher redundancy, such brain changes have a smaller impact on cognitive ability. Therefore, cognitive functioning can be preserved despite accumulating damage.

Stern and colleagues (Stern et al., 2023) suggested that three components are required for the investigation of cognitive reserve in aging: (1) measures of age-related brain changes that impact cognition; (2) measures of cognitive performance; (3) a metric that modulates/mediates the relationship between 1 and 2, i.e., the proposed metric of cognitive reserve. Two commonly reported aspects of age-related brain changes include age-related decreases in gray matter (GM) cortical volume and decreases in cortical thickness (Christova and Georgopoulos, 2023; Resnick et al., 2003; de Chastelaine et al., 2023; Steffener, 2021; Storsve et al., 2014; Fjell et al., 2009; Pfefferbaum et al., 2013; De Chastelaine et al., 2019; McGinnis et al., 2011; Salat, 2004; Bethlehem et al., 2022; Lemaitre et al., 2012). Furthermore, “brain age” may also serve as a possible metric of age-related brain changes. Several authors have developed deep learning models based on brain imaging data from healthy individuals that are able to reliably and accurately predict chronological age based upon brain-derived parameters (Kaufmann et al., 2019; Bashyam et al., 2020; Han et al., 2021; Leonardsen et al., 2021; Clausen et al., 2022; Hahn et al., 2022; Dörfel et al., 2023). These models can then be used to predict the “brain age” of participants, representing a potential biomarker of brain aging (Cole et al., 2017; Seitz-Holland et al., 2024).

There have been promising findings linking brain functional redundancy (BFR) with cognitive reserve in aging. BFR has been shown to mediate the relationship between chronological age and performance on a color-word inhibition test as an aspect of executive functioning (Sadiq et al., 2021). Furthermore, in patients with small vessel disease, BFR was reported to mediate the relationship between the presence of cerebral microbleeds and memory function assessed using an auditory verbal learning test, while other cognitive domains such as language ability or executive functioning did not show this effect (Cui et al., 2024). Together, these findings would suggest that BFR is a mechanism of cognitive reserve.

It has been posited that the neurobiological substrate of cognitive reserve is task and modality independent, supporting cognitive function as a whole (Steffener et al., 2011; Stern et al., 2018). However, whether BFR—as a mechanism of cognitive reserve—modulates the relationship between age-related brain changes and cognition across multiple cognitive domains remains an open question. In this study, we aimed to investigate whether BFR modulates the association between age-related brain changes and cognitive performance across a broad range of cognitive domains.

## 2 Materials and methods

### 2.1 Data description

**FIGURE 1:**
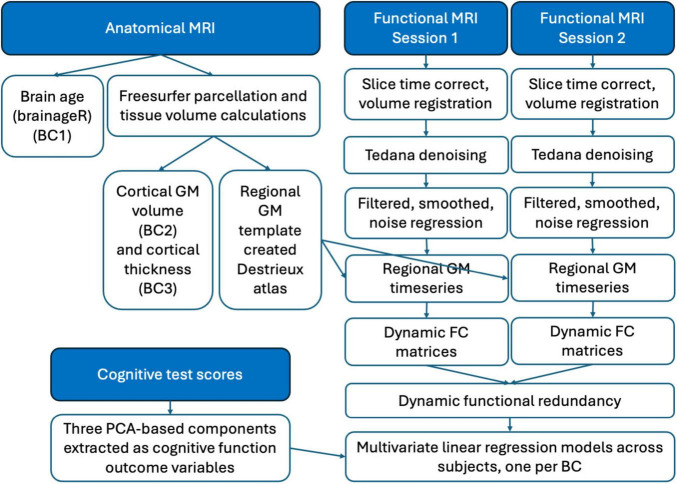
An overview of our analysis pipeline. We investigated three different definitions of age-related brain changes (BC). Brain age prediction (BC1) for each subject was calculated based upon raw anatomical MR images. Cortical grey matter (GM) volume (BC2) and cortical thickness (BC3) estimates were derived based upon the Freesurfer processing pipeline on raw anatomical MR images. Each fMRI session (*n* = 2) for each subject was individually preprocessed, from which the regional GM timeseries based upon the Destrieux GM atlas defined in Freesurfer were extracted. These regional timeseries were used to create dynamic functional connectivity (FC) matrices, from which the dynamic functional redundancy metric was calculated across both sessions. Subjects with complete cognitive test scores (*n* = 13 test scores, *n* = 260 subjects) were entered into a PCA and the scores from 3 components related to episodic, semantic and executive performance were extracted. These scores were used as outcome variables in three multivariate linear regression models, one per BC.

An open-access neuroimaging and behavioral dataset (Spreng et al., 2022) in younger- (*n* = 181; mean age 22.59 years, age range 18–34 years, 57% female) and older-aged (*n* = 120; mean age 68.63 years, age range 60-89 years, 54% female) cognitively healthy participants was utilized for this study (total beginning sample size *n* = 301). An overview of the analysis pipeline can be seen in Figure 1.

#### 2.1.1 Neuroimaging data

The resting-state functional magnetic resonance imaging data were analyzed (two sessions, lasting 20 minutes in total), as well as the anatomical T1 image acquired for each participant. The neuroimaging data were acquired across two different sites, using multi-echo imaging sequences with TR = 3,000 ms, TE = 13.7 ms/30 ms/47 ms, flip angle = 83 degrees, voxel size = 3 mm isotropic, 204 volumes/session (1 participant had 206) for site 1 and with TR = 3,000 ms, TE = 14 ms/29.96 ms/45.92 ms, flip angle = 83 degrees, voxel size = 3.4 mm × 3.4 mm × 3 mm, 200 volumes/session for site 2. Voxelwise fMRI signal can be estimated as a monoexponential decay based upon the formula (*t*) = *S*_0_e^−*t*/*T*2⁣*^. S_0_ represents the signal intensity at the point of radiofrequency excitation at time 0 and R2* = 1/T2*, where T2* is the time constant of signal decay. These two effects – S_0_ and R2* – are differentially affected by neural activity and sources of noise (e.g., head motion); neural activity influences R2* but not S_0_, whereas sources of noise alter *S*_0_ but not R2*. Therefore, sources of noise can be separated and effectively removed from fMRI signals via multi-echo imaging (Power et al., 2018).

#### 2.1.2 Behavioral data

Each participant performed an extensive test battery of various cognitive, affective and self-report measures. For our analysis, we used the following cognitive tests: Verbal Paired Associates (3 outcome variables were available for this test), Associative Recall, NIH Cognition Auditory Verbal Learning, NIH Cognition Picture Sequence Memory, Shipley Vocabulary, NIH Cognition Picture Vocabulary, NIH Cognition Oral Reading Recognition, Trail Making Task, NIH Cognition Flanker, NIH Cognition Dimensional Change Card Sort, and NIH Cognition List Sort Working Memory test.

### 2.2 Preprocessing

#### 2.2.1 Anatomical data

The three metrics of age-related brain changes were calculated from the anatomical image. The raw anatomical image for each participant was fed into the Freesurfer image analysis suite via which the cortical reconstruction and volumetric segmentation of the anatomical image was performed. This processing pipeline also generated a gray matter template based upon the Destrieux atlas (Destrieux et al., 2010) that was utilized with the fMRI data. The raw anatomical image was also analyzed using the brainageR pipeline (v2.1),^1^ shown to produce the highest accuracy and test-retest reliability in age predictions compared to other software packages (Dörfel et al., 2023). Each participant’s brain age was predicted by a model previously trained to predict the age of 3377 healthy participants (mean age = 40.6 years, SD = 21.4, age range 18–92 years) from numerous publicly available datasets based upon their anatomical data. Using normalized probability maps of cerebrospinal fluid, white matter, and gray matter, the software constructs a vector and analyses it through principal component analysis. Finally, to estimate brain age with brainageR, a Gaussian progress regression model is applied to the first 435 principal components (Dörfel et al., 2023).

#### 2.2.2 Functional data

Multi-echo fMRI data was preprocessed using AFNI, individually for each of the two sessions. The first 4 volumes were removed, and the images were then slice-time corrected. Volume registration for motion correction was first estimated on the images from the second echo and applied to all three echo images. Seven participants with greater than 2.5 mm/degree maximum displacement were excluded at this stage, leaving 294 participants remaining. The volume registered images were fed into the tedana denoising algorithm (v24.0.2; Python 3.12; Kundu et al., 2012, 2013; DuPre et al., 2021). Briefly, this pipeline uses a temporal independent component analysis on the data from all three echos to remove components in the blood-oxygenation-level dependent (BOLD) signal that are unlikely to originate from neural sources via neurovascular coupling (such as cardiac, respiratory or motion-related components). As BOLD signals follow a specific decay pattern across varying echo times (TE) due to T2* relaxation, non-BOLD components deviate from this expected behavior. Tedana evaluates these patterns using kappa and rho metrics. A high kappa value suggests that the component is likely a BOLD signal since it follows the T2*-weighted decay, whereas a high rho indicates noise. By applying a threshold based on these values, tedana separates BOLD-like signals from noise, thereby enhancing the signal-to-noise-ratio. The denoised data is then reconstructed and the three echos are optimally combined in a way that maximizes the signal-to-noise ratio across regions. The skullstripped anatomical T1 was aligned to the denoised and optimally combined functional data. The resulting transformation matrix was applied to the GM regional mask from the Freesurfer pipeline described above, providing a regional GM parcellation template in alignment with the fMRI data. This template was restricted to cortical regions only, and the mean BOLD timeseries was extracted from each cortical GM region, resulting in 167 regional timeseries per participant per session.

### 2.3 Analysis

#### 2.3.1 Anatomical data for the estimation of age-related brain changes

Estimates of GM cortical volume and cortical thickness for each participant were derived from the Freesurfer analysis pipeline. GM cortical volume was normalized to the total intracranial volume for each participant to correct for inter-participant variation in head size (Jack et al., 1992; Bobinski et al., 1999; Juottonen et al., 1999). The predicted brain age of each participant was extracted from the results of the brainageR pipeline.

#### 2.3.2 Functional data for the estimation of dynamic brain functional redundancy

##### 2.3.2.1 Creation of dynamic functional connectivity matrices

Dynamic functional connectivity matrices were created for each participant and for each session. A sliding window of 50 volumes with a step size of 1 volume was used, leading to 151 matrices per participant per session, with each matrix representing the functional connectivity (defined by the Pearson’s correlation coefficient) between all cortical GM region pairs at that window of time. Our choice of 50 volumes per window was based upon the BOLD signal frequency range commonly thought to be driven predominantly by neural activity (0.01–0.1 Hz), with slower frequencies below this range being driven by sources of noise. Given our TR of 3 s, we therefore opted for a window size of 150 s in order to capture the slowest frequencies of interest at around 0.01 Hz. Given the sensitivity of dynamic functional connectivity analyses to window size, we also ran our full analysis pipeline with a window size of 100 volumes to see whether our results were dependent on window size (see Supplementary Tables S5–S7 for the corresponding results).

##### 2.3.2.2 Brain functional redundancy estimation across time

BFR was calculated based upon the method described previously (Ghanbari et al., 2020; Ghanbari et al., 2021, 2022, 2023). Using the Brain Connectivity Toolbox (Rubinov et al., 2009), for each participant and each session, the first dynamic functional connectivity matrix was thresholded to a number of different density values (5–95% in 5% steps) and then binarized, producing 19 matrices. The number of independent pathways between each region pair was then calculated at each density threshold, starting at 5% and working up from there. When all region pairs were found to have at least one pathway between them (i.e., the network is “one-connected”), the search stopped, and the density value of this one-connected state was noted. The search then began again, only this time searching for the density at which the network is “two-connected,” with all region pairs having at least two independent pathways between them. If the density of the two-connected state was the same as the density of the one-connected state, this time window was labeled as redundant and assigned a value of 1, else 0. These steps were performed repetitively across all dynamic functional connectivity matrices, producing a vector of zeros and ones for each session. The two vectors were concatenated across sessions and the proportion of time spent in a redundant state (i.e., a value of 1) was defined as the BFR metric for each participant. Therefore, this metric defines BFR in terms of the amount of time spent in a redundant state.

#### 2.3.3 Behavioral data for the estimation of cognitive performance

The scores for 260 participants with complete data for the 13 cognitive test outcome variables were z-scored and entered into a principal component analysis using the in-built stats package within R Statistical Software (v4.1.2) (R Core Team, 2021). The variance that each component accounted for in the original data was assessed, as well as the coefficients of each component to look for any pattern regarding original categories of cognitive tests by the dataset authors—episodic, semantic and executive. The optimal number of principal components to utilize for further analysis was calculated using the find_curve_elbow function using the pathviewr package (Baliga et al., 2021) within R Statistical Software (v4.1.2) (R Core Team, 2021). This function calculates the elbow point by drawing a line between the first observation and the final observation. It then calculates the distance between each observation and that line, and the elbow of the curve is the observation that maximizes this distance. The scores of each of the chosen principal components were then entered as outcome variables in the multivariate linear regression models described below.

### 2.4 Statistical approach

Three multivariate linear regression models were created using the in-built stats package within R Statistical Software (v4.1.2) (R Core Team, 2021). Each model tested the predictive value of each of the three definitions of age-related brain changes—cortical GM volume, cortical thickness and brain age on cognitive performance. Critically, each model included BFR as a further predictor, as well as an interaction term between the age-related brain change and BFR, to test the modulating effect of BFR on the relationship between each age-related brain change and cognitive performance. Cognitive performance was defined by the scores of the chosen principal components as described above. Control variables included in the models were sex and scanning site. Chronological age was included as a further control variable in the brain age model, due to findings of a chronological age-based bias in brain age predictions (Beheshti et al., 2019; Lange and Cole, 2020; Zhang et al., 2023). As an example, the R formula for the brain age model was as follows:

mlm < - lm(cbind(PC1, PC2, PC3) ∼ redundancy + brainage + redundancy*brainage + site + sex + age, data = ageing_data)

Chronological age was not included as a control variable in the original cortical thickness and cortical GM volume models due to our expectation that chronological age and redundancy will covary with one another and lead to a masking of effects of interest in model results. For completeness, however, we have included chronological age as a further control variable in the cortical thickness and cortical GM volume models and reported these results in Supplementary Tables S3, S4.

## 3 Results

### 3.1 Principal component analysis

**FIGURE 2:**
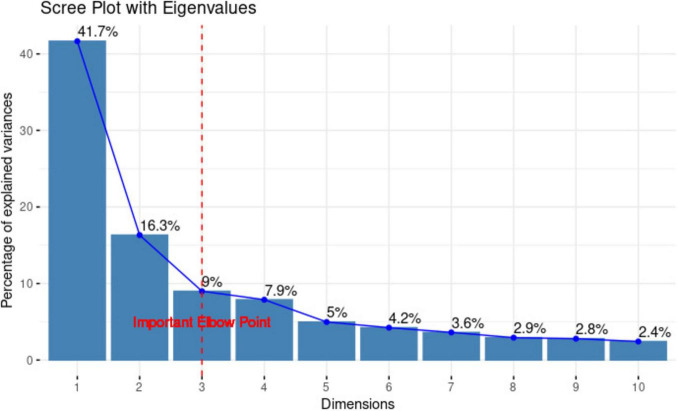
Scree plot showing the percentage of explained variance across the principal components derived from the 13 cognitive test scores across 260 subjects. Based upon the defined elbow point at component 3, scores from the first 3 components were used for statistical analysis. This figure was created using the factoextra package (Kassambara and Mundt, 2020) within R Statistical Software (v4.1.2) (R Core Team, 2021).

The results of the principal component analysis are provided in Table 1. As shown in Figure 2, the three principal components (PCs) were retained based on the identified elbow point. The first three PCs cumulatively explained 67% of the total variance in the dataset.

**TABLE 1:** Principal component coefficients for the first 4 components.

Cognitive test	PC1	PC2	PC3	PC4
Verbal paired associates: immediate recall	**0.35989061**	0.13192479	−0.30376220	0.10317762
Verbal paired associates: delayed recall	**0.36753425**	0.09831439	−0.26120144	0.09096365
Verbal paired associates: delayed free recall	**0.33431330**	0.12858128	−0.20591347	0.05286513
Associative recall	**0.35242289**	0.19333197	−0.15231261	0.10282689
NIH cognition auditory verbal learning	**0.35194963**	0.04299362	−0.00666587	−0.06897918
NIH cognition picture sequence memory	**0.32650484**	0.04835810	−0.01211713	−0.11366875
Shipley vocabulary	−0.12093573	**0.57294058**	0.08532941	0.12511439
NIH cognition picture vocabulary	−0.19122803	**0.53119811**	0.03502500	0.10400854
NIH cognition oral reading recognition	−0.09166479	**0.48648241**	0.23441390	0.01586972
Trail making task	−0.10749235	−0.09828088	−0.15573646	**0.86860715**
NIH cognition flanker	0.23465552	−0.09736644	**0.58560890**	0.30712623
NIH cognition dimensional change card sort	0.28286650	−0.14194585	**0.49455045**	0.17018009
NIH cognition list sort working memory	0.25045712	0.16866332	**0.31381856**	−0.21340988

The bold values indicate highlight results with *p* < 0.05.

Principal component 1 (PC1) was labeled as episodic memory, as it was primarily influenced by cognitive tests measuring episodic recall (PC loadings on each cognitive test can be found in Table 1). The strongest loadings were from Verbal Paired Associates with Immediate Recall, Delayed Recall and Delayed Free Recall, Associative Recall, NIH Cognition Auditory Verbal Learning, and NIH Cognition Picture Sequence Memory. It accounted for 41.7% of the total explained variance, making it the most significant contributor.

Principal component 2 (PC2), accounting for 16.3% of the variance and largely driven by Shipley Vocabulary, NIH Cognition Picture Vocabulary and NIH Cognition Oral Reading Recognition, was designated as semantic memory.

Principal component 3 (PC3) explained 9.0% of the variance. The highest coefficients were observed for the NIH Cognition Flanker, NIH Cognition Dimensional Change Card Sort and NIH Cognition List Sort Working Memory, suggesting that it reflects performance in executive functioning.

### 3.2 Multivariate linear regression models

Results of all models can be seen in detail in Tables 2–4. Below we have summarized the main findings from each model, i.e., for each definition of age-related brain change.

**TABLE 2:** Results of multivariate linear regression model across subjects with cortical thickness as age-related brain change.

Predictor	PC1: Episodic				PC2: Semantic				PC3: Executive			
	**β**	***SE***	***t***	***p***	**β**	***SE***	***t***	***p***	**β**	***SE***	***t***	***p***
Cortical thickness	**14.39**	**1.09**	**13.221**	**<0.001**	−0.87	0.90	−0.964	0.33	**1.28**	**0.64**	**1.99**	**0.047**
BFR	−**0.03**	**0.008**	−**3.40**	**<0.001**	0.01	0.006	1.81	0.07	0.008	0.005	1.95	0.052
Cortical thickness*BFR	−**0.15**	**0.06**	−**2.32**	**0.02**	0.006	0.05	0.126	0.899	0.009	0.04	0.25	0.80
Site	−**0.74**	**0.29**	−**2.60**	**0.009**	−**0.93**	**0.24**	−**3.90**	**<0.001**	**0.68**	**0.17**	**4.02**	**<0.001**
Sex	**1.02**	**0.22**	**4.71**	**<0.001**	−0.10	0.18	−0.56	0.58	−**0.40**	**0.13**	−**3.16**	**0.002**
Fit												
Res. SE	**1.71**				**1.43**				**1.01**			
R^2^	**0.47**				**0.06**				**0.15**			
Adj. R^2^	**0.46**				**0.04**				**0.13**			
F	**44.69**				**3.34**				**8.71**			
p	**<0.001**				**0.006**				**<0.001**			

The bold values indicate highlight results with *p* < 0.05.

**TABLE 3:** Results of multivariate linear regression model across subjects with GM volume as age-related brain change.

Predictor	PC1: Episodic				PC2: Semantic				PC3: Executive			
	**β**	***SE***	***t***	***p***	**β**	***SE***	***t***	***p***	**β**	***SE***	***t***	***p***
GM volume	**59.65**	**4.64**	**12.87**	**<0.001**	−7.28	3.79	−1.92	0.06	**5.62**	**2.70**	**2.08**	**0.04**
BFR	−**0.02**	**0.008**	−**2.799**	**0.006**	0.01	0.007	1.86	0.06	0.008	0.005	1.69	0.09
GM volume*BFR	−0.30	0.32	−0.94	0.35	0.19	0.27	0.71	0.47	−0.08	0.19	−0.42	0.67
Site	−0.46	0.29	−1.60	0.11	−**0.95**	**0.24**	−**4.03**	**<0.001**	**0.70**	**0.17**	**4.17**	**<0.001**
Sex	**0.51**	**0.22**	**2.33**	**0.02**	−0.04	0.18	−0.22	0.82	−**0.45**	**0.13**	−**3.55**	**<0.001**
Fit												
Res. SE	**1.73**				**1.42**				**1.01**			
R^2^	**0.46**				**0.07**				**0.15**			
Adj. R^2^	**0.45**				**0.06**				**0.13**			
F	**42.67**				**4.02**				**8.72**			
p	**<0.001**				**0.002**				**<0.001**			

The bold values indicate highlight results with *p* < 0.05.

**TABLE 4:** Results of multivariate linear regression model across subjects with brain age as age-related brain change.

Predictor	PC1: Episodic				PC2: Semantic				PC3: Executive			
	**β**	***SE***	***t***	***p***	**β**	***SE***	***t***	***p***	**β**	***SE***	***t***	***p***
Brain age	−0.02	0.016	−1.41	0.16	−0.02	0.02	−1.26	0.21	−0.01	0.01	−0.52	0.60
BFR	−0.004	0.006	−0.67	0.503	0.004	0.01	0.55	0.58	0.01	0.01	1.61	0.11
Brain age*BFR	−0.00003	−0.0002	−0.13	0.89	0.0001	0.0002	0.47	0.64	0.0001	0.0002	0.62	0.54
Site	**0.81**	**0.16**	**5.1**	**<0.001**	−**1.003**	**0.24**	−**4.25**	**<0.001**	**0.75**	**0.17**	**4.37**	**<0.001**
Sex	−**0.06**	**0.02**	−**3.79**	**<0.001**	−0.10	0.17	−0.56	0.57	−**0.43**	**0.13**	−**3.40**	**<0.001**
Age	−**0.06**	**0.02**	−**3.79**	**<0.001**	**0.04**	**0.02**	**2.17**	**0.03**	−0.001	0.01	−0.12	0.91
Fit												
Res. SE	**1.27**				**1.38**				**1.002**			
R^2^	**0.71**				**0.12**				**0.16**			
Adj. R^2^	**0.70**				**0.10**				**0.14**			
F	**102.9**				**6.004**				**8.03**			
p	**<0.001**				**<0.001**				**<0.001**			

The bold values indicate highlight results with *p* < 0.05.

#### 3.2.1 Model 1: age-related brain changes as defined by cortical thickness

**FIGURE 3:**
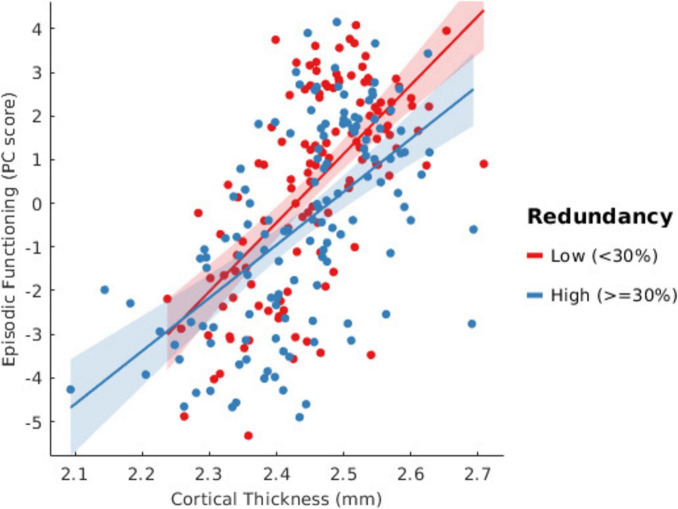
Scatterplot showing the association between brain functional redundancy and episodic functioning across groups of participants with “low” versus “high” cortical thickness. Participants with a cortical thickness value greater than or equal to the mean value were assigned to the “high” group, and those with a value less than the mean were assigned to the “low” group.

As an aspect of age-related brain change, cortical thickness was significantly predicted by the first principal component representing episodic memory function (β = 14.39, SE = 1.09, *t* = 13.221, *p* < 0.001). BFR was a significant predictor of episodic functioning, displaying a negative association (β = −0.03, SE = 0.008, *t* = −3.40, *p* < 0.001), suggesting that higher BFR is related to worse episodic performance. Figure 3 displays the relationship between BFR and episodic functioning within two subgroups of age-related brain change: “low” vs. “high” cortical thickness. Participants with a cortical thickness value greater than or equal to the mean value were assigned to the “high” group, and those with a value less than the mean were assigned to the “low” group. Across both subgroups, a common generally negative association between BFR and episodic functioning can be seen. Therefore, we assume that the directions of association as reported at the population level are not attributable to Simpson’s paradox (Cabeza et al., 2018).

**FIGURE 4:**
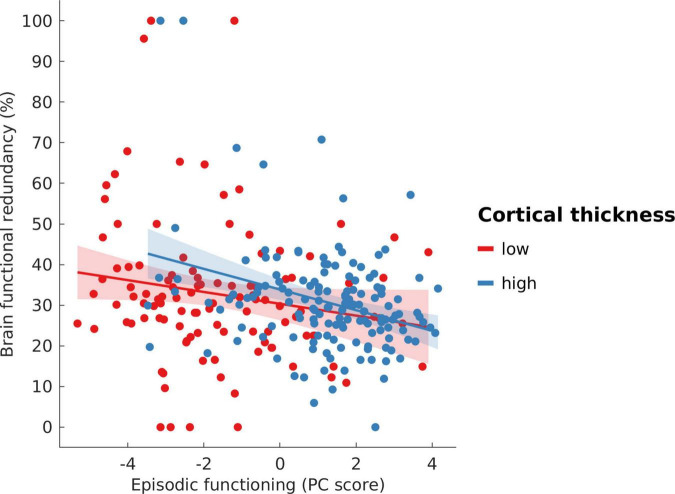
Scatterplot showing the association between cortical thickness and episodic functioning across subjects. Subjects were split according to low or high brain functional redundancy (BFR). A threshold of 30% was chosen to derive the two groups to achieve an approximately equal number of subjects in each group (Low *n* = 128; High *n* = 132). A linear fit for each group displays a steeper slope for the low redundancy group compared with the high redundancy group; subjects with high BFR show a lower impact of changes in cortical thickness on episodic functioning as compared with subjects with low BFR.

Furthermore, a significant interaction between cortical thickness and BFR was found for episodic functioning (β = −0.15, SE = 0.06, *t* = −2.32, *p* = 0.02). This indicates a diminishing positive effect of cortical thickness on episodic performance as BFR increases (as displayed in Figure 4). Among the covariates, site (β = −0.74, SE = 0.29, *t* = −2.60, *p* = 0.009) and sex (β = 1.02, SE = 0.22, *t* = 4.71, *p* < 0.001) were also significant predictors. When chronological age was included as a confound variable in the model, this negative association as well as the interaction effect were no longer significant (see Supplementary Table S3).

As displayed in Table 1, for the second principal component (semantic functioning), neither cortical thickness nor BFR significantly predicted performance. There was also no significant interaction, implying that the relationship between cortical thickness and semantic memory is not moderated by BFR. Site was the only significant predictor in this model (β = −0.93, SE = 0.24, *t* = −3.90, *p* < 0.001), indicating variability across different study sites.

The third principal component (executive functioning) showed a significant association with cortical thickness (β = 1.28, SE = 0.64, *t* = 1.99, *p* = 0.047), with greater cortical thickness predicting better executive performance. However, there was neither a significant predictive effect of BFR nor a significant interaction between cortical thickness and BFR considering executive functioning. Both site (β = 0.68, SE = 0.17, *t* = 4.02, *p* < 0.001) and sex (β = −0.40, SE = 0.13, *t* = −3.16, *p* = 0.002) were significant predictors.

#### 3.2.2 Model 2: age-related brain changes as defined by GM volume

**FIGURE 5:**
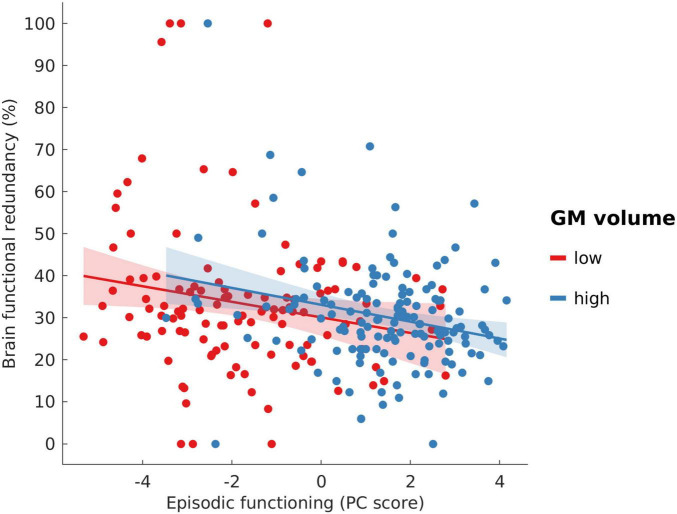
Scatterplot showing the association between brain functional redundancy and episodic functioning across groups of participants with “low” versus “high” cortical GM volume. Participants with a cortical GM volume value greater than or equal to the mean value were assigned to the “high” group, and those with a value less than the mean were assigned to the “low” group.

As a second aspect of age-related brain changes, GM volume exhibited a highly significant positive predictive effect on episodic functioning (β = 59.65, SE = 4.64, *t* = 12.87, *p* <0.001), suggesting an association between greater GM volume and better episodic performance. Further, a significant negative relationship between BFR and episodic functioning was observed (β = −0.02, SE = 0.008, *t* = −2.80, *p* = 0.006) with higher BFR predicting worse episodic performance. Figure 5 displays the relationship between BFR and episodic functioning within two subgroups of age-related brain change: “low” vs. “high” cortical GM volume (defined the same way as described above in section 3.2.1). Across both subgroups, a negative association between BFR and episodic functioning can be seen. When chronological age was included as a confound variable in the model, this negative association was no longer significant (see Supplementary Table S4). As shown in Table 2, there was no significant interaction effect between GM volume and BFR in predicting episodic functioning, indicating that BFR does not have a moderating effect. Moreover, considering the covariates, sex was a significant predictor (β = 0.51, SE = 0.22, *t* = 2.33, *p* = 0.02).

However, for semantic functioning, neither GM volume nor BFR significantly predicted performance. As displayed in Table 2, there was also no moderating effect of BFR. The covariate site was a significant predictor in this model (β = −0.95, SE = 0.24, *t* = −4.03, *p* < 0.001).

With greater GM volume predicting better executive performance, GM volume was significantly associated with the third principal component (β = 5.62, SE = 2.70, *t* = 2.08, *p* = 0.04). BFR had no predictive or moderating effect. Both site (β = 0.70, SE = 0.17, *t* = 4.17, *p* < 0.001) and sex (β = −0.45, SE = 0.13, *t* = −3.55, *p* < 0.001) were significant predictors for executive functioning.

#### 3.2.3 Model 3: age-related brain changes as defined by brain age

Brain age, as our third aspect of age-related brain changes, did not significantly predict episodic functioning, as displayed in Table 3. Similarly, BFR showed no predictive or moderating effect on the first principal component. Again both, site (β = 0.81, SE = 0.16, *t* = 5.10, *p* < 0.001) and sex (β = 0.06, SE = 0.02, *t* = −3.79, *p* < 0.001), were significant predictors. Additionally, chronological age had a significant negative effect on episodic performance in this model (β = −0.06, SE = 0.02, *t* = −3.79, *p* < 0.001).

Neither brain age nor BFR had a predictive effect on semantic performance, and there was no significant interaction between brain age and BFR. Site remained the only significant predictor among the covariates for the second principal component (β = −1.003, SE = 0.24, *t* = −4.25, *p* < 0.001).

For executive function, there was no predictive effect of brain age or BFR. Further, BFR did not show a moderating effect. However, both site (β = 0.75, SE = 0.17, *t* = 4.37, *p* < 0.001) and sex (β = −0.43, SE = 0.13, *t* = −3.40, *p* < 0.001) were significant predictors, while chronological age did not have a significant impact on the executive performance.

## 4 Discussion

This study aimed to investigate whether the time spent in a redundant state within functional brain networks, referred to as brain functional redundancy (BFR), serves as a mechanism of cognitive reserve providing an explanation for a person’s resiliency to age-related brain changes in terms of their cognitive abilities across multiple cognitive domains. We utilized three definitions of age-related brain changes and investigated whether BFR modulates the relationship between these brain changes and cognitive performance across three domains—executive, semantic, and episodic—in a cross-sectional cohort of participants across the lifespan.

Increased BFR was predictive of reduced performance in episodic functioning in both the cortical thickness and GM volume models. This could be interpreted to indicate a compensatory mechanism in the face of age-related brain changes. One could argue that a reduced performance with increasing BFR cannot be indicative of a compensatory process, however, it may also be possible that BFR acts to keep the cognitive domain “afloat,” i.e., maintain a certain level of functioning, even if this level of functioning is not necessarily optimal. A similar observation has been previously reported (Zarahn et al., 2007), where older participants were found to recruit additional brain regions beyond certain “primary” activated regions during execution of a demanding cognitive task as compared with younger participants. Interestingly, recruitment of these additional brain regions was not only related to atrophy in the primary regions (Steffener et al., 2009) but was also associated with reduced task performance. It seemed that the activation of these additional regions served as a compensatory mechanism, with the goal of maintaining function in the face of age-related brain atrophy—with maintenance rather than improvement of function being key here.

We identified a modulating effect of BFR on the relationship between cortical thickness and episodic functioning. For every unit increase in BFR, there was a reduced effect of cortical thickness on episodic functioning. This finding would be in line with BFR as a metric of cognitive reserve: with increased functional brain resiliency driven by increased BFR, changes in cortical thickness have less of an impact on episodic functioning. Participants with higher BFR, developed as a compensation strategy in the face of age-related brain changes marked by decreased cortical thickness, may be more resilient to subsequent changes in episodic functioning.

Noteworthy, however, is the fact that this modulating effect was no longer significant when using a longer window size (100 volumes as opposed to the original choice of 50 volumes) for the creation of the dynamic functional connectivity matrices on which the BFR metric was calculated (see Supplementary Table S5). As BFR is a metric reflecting dynamics across time, a window size of 100 volumes with a TR of 3 seconds might not be able to adequately capture changes in redundancy, possibly leading to more inaccurate estimates of BFR. We would argue that a window size of 50 volumes is a more logical choice, with a shorter timespan to better capture temporal dynamics whilst also being long enough to capture slow BOLD frequencies of interest. This modulating effect, as well as the predictive value of BFR on episodic functioning in both cortical thickness and cortical GM volume models, was no longer significant following the inclusion of chronological age as a confounder in the regression models. It is difficult to say whether the significant effects were predominantly driven by chronological age or whether BFR plays a true modulating role on the effect of cortical thickness on episodic functioning that we can no longer “uncover” when including chronological age as a covariate.

When defining age-related brain changes based upon cortical GM volume or brain age, this modulating effect was no longer present. Measures of cortical thickness and cortical GM volume both identify morphometric changes, but with only partially overlapping regions. Discrepancies between the two measures have been found to be due to regional variations in surface area, curvature and gray/white matter intensity contrast (Kong et al., 2015). How the modulating effect of BFR depends on these morphometric differences warrants further investigation. Brain age shows promise as a biomarker for ageng. However, a number of open questions remain regarding this metric and requires further optimization due to its high susceptibility to low image quality and motion (Hanson et al., 2024), which unfortunately commonly occurs in older-aged participants. The bias that occurs in the predicted brain age based upon the chronological age of the participant is also not a problem that has been solved: the best practice is to currently either use a regression approach or include chronological age as a covariate in order to correct for this bias, but this has also been shown to have its own limitations (Butler et al., 2021). We included brain age as a potentially interesting metric of age-related brain changes but suggest to interpret these findings with caution.

Interestingly these effects were also only observed within the episodic domain; there were no predictive effects of BFR on semantic or executive functioning, nor were there any modulating effects of BFR on the associations between age-related brain changes and semantic or executive functioning. Decline in episodic memory can be seen as one of the early cognitive changes across the adult lifespan, with an accelerating deterioration occurring in older age. Many studies have observed different activation patterns in additional compensatory networks in response to this episodic memory decline (Nyberg et al., 2012; Tromp et al., 2015). According to our findings, BFR could potentially be a metric of cognitive reserve in aging specifically regarding episodic functioning. This would argue against the idea that cognitive reserve may be supported by a mechanism that generalizes across multiple cognitive domains, but rather there might be multiple underlying mechanisms of cognitive reserve in aging that support different aspects of cognition in the face of age-related brain changes.

### 4.1 Strengths and limitations

The participants were in the resting state with eyes closed during fMRI data acquisition. Although the dynamic functional organization of the brain observed during the resting state closely parallels that observed during the task state, there are still some significant functional organizational changes that occur during the task state that contribute to task performance (Cole et al., 2021). Whether the domain specificity of BFR as a metric of cognitive reserve is different across rest or task states is an interesting and open question. At this point it is also worth further noting that the sample consisted of healthy participants, which may have resulted in too little variance to effectively demonstrate domain-general cognitive reserve. The findings of BFR as a mediator of the relationship between chronological age and executive functioning in Sadiq et al. was based upon fMRI data during the resting state. However these authors used a different metric of BFR based upon the static functional connectome than the one utilized here (Sadiq et al., 2021). There is considerable variation across studies in the operationalization of redundancy, as well as the definition of age-related brain changes, with limitations attributable to each. Here we chose to utilize a metric based upon the dynamic nature of brain functional connectivity, coming with it the limitation of subjective methodological choices such as the window size used for the creation of the dynamic functional connectivity matrices. The dependency of our results on window size and the exclusion of chronological age as a confound variable represents a limitation of this method and it would be worth investigating whether this modulating role exists when using alternative metrics of redundancy not dependent on such methodological choices.

Most previous work has been conducted on cross-sectional cohorts, a limitation of the current work, seeing as a longitudinal design would be much better suited to the identification of mechanisms that drive resiliency to brain changes that occur with aging (Pettigrew and Soldan, 2019). Furthermore, the predetermined selection of cognitive tests included only a small number of cognitive domains. It is questionable how much variance they accounted for, representing another limitation of the present study.

While our results provide valuable insights, the inclusion of more diverse participant groups could further enhance the generalizability of our findings. An interesting area for future research is the influence of geographical factors, e.g., differences between rural or urban living, on aging and cognitive reserve. Urban environments often offer greater access to educational opportunities, diverse social networks, and cognitive stimulation, which are known to contribute significantly to building cognitive reserve. Conversely, rural settings, despite challenges such as limited access to healthcare and cognitive engagement opportunities, may provide cognitive resilience through closer, stronger social ties and lower levels of environmental stressors like noise and pollution (Cassarino and Setti, 2015). Studies have found that urban residents often perform better on a broad range of cognitive tasks, for example those measuring memory skills, than their rural counterparts, likely due to differences in educational attainment and occupational complexity (Linnell et al., 2013; Jokela, 2014; Cassarino et al., 2018; Saenz et al., 2018, 2022; Lawrence et al., 2023; Steinberg et al., 2023). However, an overstimulating urban environment may negatively affect attention control (Cassarino and Setti, 2015). Additionally, the predominance of white participants in the dataset used highlights another limitation of the current study. While research has explored sex or gender differences in specific contributors to cognitive reserve (Letenneur et al., 2000; Beinhoff et al., 2009; Sundermann et al., 2016; Malpetti et al., 2017; Santabárbara et al., 2019), there is a lack of studies examining these differences within cognitive reserve itself (Subramaniapillai et al., 2021). For instance, men tend to exhibit a larger absolute GM volume, while women have thicker cortices and greater GM volumes when adjusting for skull size (Arenaza-Urquijo et al., 2024). Differences in glucose metabolism functional network activation patterns may also play a role (Subramaniapillai et al., 2021; Arenaza-Urquijo et al., 2024). Future research could provide a more comprehensive understanding of cognitive reserve in aging across diverse populations—particularly in the context of evolving societies, fluid gender norms, and increasing urbanization.

## 5 Conclusion

BFR could potentially serve as a metric of cognitive reserve in aging that supports the maintenance of episodic functioning in the face of age-related changes in cortical thickness. Future research should look to systematically investigate the effect that methodological differences across previous studies have on the interpretation of BFR as a cognitive domain-specific or domain-general metric of cognitive reserve in aging.

## Data Availability

Publicly available datasets were analyzed in this study. This data can be found at: https://openneuro.org/datasets/ds003592/versions/1.0.13 (Spreng et al., 2022).

## Footnotes

1
https://osf.io/4ngr8

## References

[B1] Anthony M. Lin F. (2017). A systematic review for functional neuroimaging studies of cognitive reserve across the cognitive aging spectrum. *Arch. Clin. Neuropsychol.* 33 937–948. 10.1093/arclin/acx125 PMC6246379 29244054

[B2] Arenaza-Urquijo E. M. Boyle R. Casaletto K. Anstey K. J. Vila-Castelar C. Colverson A. (2024). Sex and gender differences in cognitive resilience to aging and Alzheimer’s disease. *Alzheimers Dement. J. Alzheimers Assoc.* 20 5695–5719. 10.1002/alz.13844 38967222 PMC11350140

[B3] Baliga V. B. Armstrong M. S. Press E. R. (2021). *pathviewr: Tools to import, clean, and visualize animal movement data in R. R package version 1.1.7.* 10.5281/zenodo.4270187

[B4] Bashyam V. M. Erus G. Doshi J. Habes M. Nasrallah I. M. Truelove-Hill M. (2020). MRI signatures of brain age and disease over the lifespan based on a deep brain network and 14 468 individuals worldwide. *Brain* 143 2312–2324. 10.1093/brain/awaa160 32591831 PMC7364766

[B5] Beheshti I. Nugent S. Potvin O. Duchesne S. (2019). Bias-adjustment in neuroimaging-based brain age frameworks: A robust scheme. *NeuroImage Clin.* 24:102063. 10.1016/j.nicl.2019.102063 31795063 PMC6861562

[B6] Beinhoff U. Tumani H. Riepe M. W. (2009). Applying new research criteria for diagnosis of early Alzheimer’s disease: Sex and intelligence matter. *Int. J. Alzheimers Dis.* 2009:638145. 10.4061/2009/638145 20798761 PMC2925096

[B7] Bennett D. A. Wilson R. S. Schneider J. A. Evans D. A. Mendes, de Leon C. F. (2003). Education modifies the relation of AD pathology to level of cognitive function in older persons. *Neurology* 60 1909–1915. 10.1212/01.wnl.0000069923.64550.9f 12821732

[B8] Bethlehem R. A. I. Seidlitz J. White S. R. Vogel J. W. Anderson K. M. Adamson C. (2022). Brain charts for the human lifespan. *Nature* 604 525–533. 10.1038/s41586-022-04554-y 35388223 PMC9021021

[B9] Bobinski M. de Leon M. J. Convit A. Santi S. D. Wegiel J. Tarshish C. Y. (1999). MRI of entorhinal cortex in mild Alzheimer’s disease. *Lancet* 353 38–40. 10.1016/S0140-6736(05)74869-8 10023955

[B10] Butler E. R. Chen A. Ramadan R. Le T. T. Ruparel K. Moore T. M. (2021). Pitfalls in brain age analyses. *Hum. Brain Mapp.* 42 4092–4101. 10.1002/hbm.25533 34190372 PMC8357007

[B11] Cabeza R. Albert M. Belleville S. Craik F. I. M. Duarte A. Grady C. L. (2018). Maintenance, reserve and compensation: The cognitive neuroscience of healthy ageing. *Nat. Rev. Neurosci.* 19 701–710. 10.1038/s41583-018-0068-2 30305711 PMC6472256

[B12] Cassarino M. O’Sullivan V. Kenny R. A. Setti A. (2018). Disabilities moderate the association between neighbourhood urbanity and cognitive health: Results from the Irish longitudinal study on ageing. *Disabil. Health J.* 11 359–366. 10.1016/j.dhjo.2017.12.002 29269303

[B13] Cassarino M. Setti A. (2015). Environment as “Brain Training”: A review of geographical and physical environmental influences on cognitive ageing. *Ageing Res. Rev.* 23 167–182. 10.1016/j.arr.2015.06.003 26144974

[B14] Christova P. Georgopoulos A. P. (2023). Changes of cortical gray matter volume during development: A human connectome project study. *J. Neurophysiol.* 130 117–122. 10.1152/jn.00164.2023 37314080 PMC10312313

[B15] Clausen A. N. Fercho K. A. Monsour M. Disner S. Salminen L. Haswell C. C. (2022). Assessment of brain age in posttraumatic stress disorder: Findings from the ENIGMA PTSD and brain age working groups. *Brain Behav.* 12:e2413. 10.1002/brb3.2413 34907666 PMC8785613

[B16] Cole J. H. Poudel R. P. K. Tsagkrasoulis D. Caan M. W. A. Steves C. Spector T. D. (2017). Predicting brain age with deep learning from raw imaging data results in a reliable and heritable biomarker. *Neuroimage* 163 115–124. 10.1016/j.neuroimage.2017.07.059 28765056

[B17] Cole M. W. Ito T. Cocuzza C. Sanchez-Romero R. (2021). The functional relevance of task-state functional connectivity. *J. Neurosci.* 41:2684. 10.1523/JNEUROSCI.1713-20.2021 33542083 PMC8018740

[B18] Crystal H. Dickson D. Fuld P. Masur D. Scott R. Mehler M. (1988). Clinico-pathologic studies in dementia: Nondemented subjects with pathologically confirmed Alzheimer’s disease. *Neurology* 38 1682–1687. 10.1212/wnl.38.11.1682 3185902

[B19] Cui L. Hong H. Wang S. Zeng Q. Jiaerken Y. Yu X. (2024). Small vessel disease and cognitive reserve oppositely modulate global network redundancy and cognitive function: A study in middle-to-old aged community participants. *Hum. Brain Mapp.* 45:e26634. 10.1002/hbm.26634 38553856 PMC10980841

[B20] De Chastelaine M. Donley B. E. Kennedy K. M. Rugg M. D. (2019). Age moderates the relationship between cortical thickness and cognitive performance. *Neuropsychologia* 132:107136. 10.1016/j.neuropsychologia.2019.107136 31288025 PMC6702041

[B21] de Chastelaine M. Srokova S. Hou M. Kidwai A. Kafafi S. S. Racenstein M. L. (2023). Cortical thickness, gray matter volume, and cognitive performance: A crosssectional study of the moderating effects of age on their interrelationships. *Cereb. Cortex N. Y. N.* 33 6474–6485. 10.1093/cercor/bhac518 36627250 PMC10183746

[B22] Destrieux C. Fischl B. Dale A. Halgren E. (2010). Automatic parcellation of human cortical gyri and sulci using standard anatomical nomenclature. *NeuroImage* 53 1–15. 10.1016/j.neuroimage.2010.06.010 20547229 PMC2937159

[B23] Dörfel R. P. Arenas-Gomez J. M. Fisher P. M. Ganz M. Knudsen G. M. Svensson J. E. (2023). Prediction of brain age using structural magnetic resonance imaging: A comparison of accuracy and test–retest reliability of publicly available software packages. *Hum. Brain Mapp.* 44 6139–6148. 10.1002/hbm.26502 37843020 PMC10619370

[B24] DuPre E. Salo T. Ahmed Z. Bandettini P. A. Bottenhorn K. L. Caballero-Gaudes C. (2021). TE-dependent analysis of multi-echo fMRI with *tedana*. *J. Open Source Softw.* 6:3669. 10.21105/joss.03669

[B25] Fjell A. M. Walhovd K. B. Fennema-Notestine C. McEvoy L. K. Hagler D. J. Holland D. (2009). One-year brain atrophy evident in healthy aging. *J. Neurosci.* 29 15223–15231. 10.1523/JNEUROSCI.3252-09.2009 19955375 PMC2827793

[B26] Franzmeier N. Buerger K. Teipel S. Stern Y. Dichgans M. Ewers M. (2017). Cognitive reserve moderates the association between functional network anti-correlations and memory in MCI. *Neurobiol. Aging* 50 152–162. 10.1016/j.neurobiolaging.2016.11.013 28017480

[B27] Ghanbari M. Hsu L. Zhou Z. Ghanbari A. Mo Z. Yap P Zhang H. Shen D. (2020). *A New Metric for Characterizing Dynamic Redundancy of Dense Brain Chronnectome and Its Application to Early Detection of Alzheimer’s Disease.* (Springer, Cham), 3–12.

[B28] Ghanbari M. Li G. Hsu L. M. Yap P. T. (2023). Accumulation of network redundancy marks the early stage of Alzheimer’s disease. *Hum Brain Mapp.* 44 2993–3006. 10.1002/hbm.26257 36896755 PMC10171535

[B29] Ghanbari M. Soussia M. Jiang W. Wei D. Yap P. T. Shen D. (2022). Alterations of dynamic redundancy of functional brain subnetworks in Alzheimer’s disease and major depression disorders. *Neuroimage Clin.* 33:102917. 10.1016/j.nicl.2021.102917 34929585 PMC8688702

[B30] Ghanbari M. Zhou Z. Hsu L. M. Han Y. Sun Y. Yap P. T. (2021). Altered connectedness of the brain chronnectome during the progression to Alzheimer’s disease. *Neuroinformatics* 20 391–403. 10.1007/s12021-021-09554-3 34837154

[B31] Hahn T. Ernsting J. Winter N. R. Holstein V. Leenings R. Beisemann M. (2022). An uncertainty-aware, shareable, and transparent neural network architecture for brain-age modeling. *Sci. Adv.* 8:eabg9471. 10.1126/sciadv.abg9471 34985964 PMC8730629

[B32] Han L. K. M. Dinga R. Hahn T. Ching C. R. K. Eyler L. T. Aftanas L. (2021). Brain aging in major depressive disorder: Results from the ENIGMA major depressive disorder working group. *Mol. Psychiatry* 26 5124–5139. 10.1038/s41380-020-0754-0 32424236 PMC8589647

[B33] Hanson J. L. Adkins D. J. Bacas E. Zhou P. (2024). Examining the reliability of brain age algorithms under varying degrees of participant motion. *Brain Inform.* 11:9. 10.1186/s40708-024-00223-0 38573551 PMC10994881

[B34] Holtzer R. Rakitin B. C. Steffener J. Flynn J. Kumar A. Stern Y. (2009). Age effects on load-dependent brain activations in working memory for novel material. *Brain Res.* 1249 148–161. 10.1016/j.brainres.2008.10.009 18983833 PMC2677982

[B35] Jack C. R. Petersen R. C. O’Brien P. C. Tangalos E. G. (1992). MR-based hippocampal volumetry in the diagnosis of Alzheimer’s disease. *Neurology* 42 183–188. 10.1212/wnl.42.1.183 1734300

[B36] Jokela M. (2014). Flow of cognitive capital across rural and urban United States. *Intelligence* 46 47–53. 10.1016/j.intell.2014.05.003

[B37] Juottonen K. Laakso M. P. Partanen K. Soininen H. (1999). Comparative MR analysis of the entorhinal cortex and hippocampus in diagnosing Alzheimer disease. *AJNR Am. J. Neuroradiol.* 20 139–144. 9974069

[B38] Kassambara A. Mundt F. (2020). *factoextra: Extract and Visualize the Results of Multivariate Data Analyses. R package version 1.0.7.* Available online at: https://cran.r-project.org/web/packages/factoextra/index.html (accessed November 21, 2024).

[B39] Katzman R. Terry R. DeTeresa R. Brown T. Davies P. Fuld P. (1988). Clinical, pathological, and neurochemical changes in dementia: A subgroup with preserved mental status and numerous neocortical plaques. *Ann. Neurol.* 23 138–144. 10.1002/ana.410230206 2897823

[B40] Kaufmann T. van der Meer D. Doan N. T. Schwarz E. Lund M. J. Agartz I. (2019). Common brain disorders are associated with heritable patterns of apparent aging of the brain. *Nat. Neurosci.* 22 1617–1623. 10.1038/s41593-019-0471-7 31551603 PMC6823048

[B41] Kong L. Herold C. J. Zöllner F. Salat D. H. Lässer M. M. Schmid L. A. (2015). Comparison of grey matter volume and thickness for analysing cortical changes in chronic schizophrenia: A matter of surface area, grey/white matter intensity contrast, and curvature. *Psychiatry Res.* 231 176–183. 10.1016/j.pscychresns.2014.12.004 25595222

[B42] Kundu P. Brenowitz N. D. Voon V. Worbe Y. Vértes P. E. Inati S. J. (2013). Integrated strategy for improving functional connectivity mapping using multiecho fMRI. *Proc. Natl. Acad. Sci. U. S. A.* 110 16187–16192. 10.1073/pnas.1301725110 24038744 PMC3791700

[B43] Kundu P. Inati S. J. Evans J. W. Luh W.-M. Bandettini P. A. (2012). Differentiating BOLD and non-BOLD signals in fMRI time series using multi-echo EPI. *NeuroImage* 60 1759–1770. 10.1016/j.neuroimage.2011.12.028 22209809 PMC3350785

[B44] Lange A. Cole J. H. (2020). Commentary: Correction procedures in brain-age prediction. *NeuroImage Clin.* 26:102229. 10.1016/j.nicl.2020.102229 32120292 PMC7049655

[B45] Lawrence E. John S. E. Bhatta T. (2023). Urbanicity and cognitive functioning in later life. *Alzheimers Dement. Diagn. Assess. Dis. Monit.* 15:e12429. 10.1002/dad2.12429 37124156 PMC10130675

[B46] Lemaitre H. Goldman A. L. Sambataro F. Verchinski B. A. Meyer-Lindenberg A. Weinberger D. R. (2012). Normal age-related brain morphometric changes: Nonuniformity across cortical thickness, surface area and gray matter volume? *Neurobiol. Aging* 33:617.e1-9. 10.1016/j.neurobiolaging.2010.07.013 20739099 PMC3026893

[B47] Leonardsen E. H. Peng H. Kaufmann T. Agartz I. Andreassen O. A. Celius E. G. (2021). Deep neural networks learn general and clinically relevant representations of the ageing brain. *Neuroimage* 256:119210. 10.1101/2021.10.29.21265645 PMC7614754 35462035

[B48] Letenneur L. Launer J. Andersen K. Dewey M. E. Ott A. Copeland J. R. M. (2000). Education and risk for Alzheimer’s disease: Sex makes a difference EURODEM pooled analyses. *Am. J. Epidemiol.* 151 1064–1071. 10.1093/oxfordjournals.aje.a010149 10873130

[B49] Linnell K. J. Caparos S. de Fockert J. W. Davidoff J. (2013). Urbanization decreases attentional engagement. *J. Exp. Psychol. Hum. Percept. Perform.* 39 1232–1247. 10.1037/a0031139 23339348

[B50] Malpetti M. Ballarini T. Presotto L. Garibotto V. Tettamanti M. Perani D. (2017). Gender differences in healthy aging and Alzheimer’s Dementia: A 18 F-FDG-PET study of brain and cognitive reserve. *Hum. Brain Mapp.* 38 4212–4227. 10.1002/hbm.23659 28561534 PMC6866811

[B51] McGinnis S. M. Brickhouse M. Pascual B. Dickerson B. C. (2011). Age-related changes in the thickness of cortical zones in humans. *Brain Topogr.* 24 279–291. 10.1007/s10548-011-0198-6 21842406 PMC3600370

[B52] Morris J. C. Storandt M. McKeel D. W. Rubin E. H. Price J. L. Grant E. A. (1996). Cerebral amyloid deposition and diffuse plaques in “normal” aging: Evidence for presymptomatic and very mild Alzheimer’s disease. *Neurology* 46 707–719. 10.1212/wnl.46.3.707 8618671

[B53] Mortimer J. A. Snowdon D. A. Markesbery W. R. (2003). Head circumference, education and risk of dementia: Findings from the Nun Study. *J. Clin. Exp. Neuropsychol.* 25 671–679. 10.1076/jcen.25.5.671.14584 12815504

[B54] Nyberg L. Lövdén M. Riklund K. Lindenberger U. Bäckman L. (2012). Memory aging and brain maintenance. *Trends Cogn. Sci.* 16 292–305. 10.1016/j.tics.2012.04.005 22542563

[B55] Pettigrew C. Soldan A. (2019). Defining cognitive reserve and implications for cognitive aging. *Curr. Neurol. Neurosci. Rep.* 19:1. 10.1007/s11910-019-0917-z 30627880 PMC7812665

[B56] Pfefferbaum A. Rohlfing T. Rosenbloom M. J. Chu W. Colrain I. M. Sullivan E. V. (2013). Variation in longitudinal trajectories of regional brain volumes of healthy men and women (ages 10 to 85years) measured with atlas-based parcellation of MRI. *NeuroImage* 65 176–193. 10.1016/j.neuroimage.2012.10.008 23063452 PMC3516371

[B57] Power J. D. Plitt M. Gotts S. J. Kundu P. Voon V. Bandettini P. A. (2018). Ridding fMRI data of motion-related influences: Removal of signals with distinct spatial and physical bases in multiecho data. *Proc. Natl. Acad. Sci. U. S. A.* 115 E2105–E2114. 10.1073/pnas.1720985115 29440410 PMC5834724

[B58] Price J. L. Morris J. C. (1999). Tangles and plaques in nondemented aging and “preclinical” Alzheimer’s disease. *Ann. Neurol.* 45 358–368. 10.1002/1531-8249(199903)45:3 10072051

[B59] R Core Team (2021). *R: A Language and Environment for Statistical Computing.* Vienna: R Foundation for Statistical Computing.

[B60] Resnick S. M. Pham D. L. Kraut M. A. Zonderman A. B. Davatzikos C. (2003). Longitudinal magnetic resonance imaging studies of older adults: A shrinking brain. *J. Neurosci.* 23 3295–3301. 10.1523/JNEUROSCI.23-08-03295.2003 12716936 PMC6742337

[B61] Reuter-Lorenz P. A. Park D. C. (2014). How does it STAC Up? revisiting the scaffolding theory of aging and cognition. *Neuropsychol. Rev.* 24:355. 10.1007/s11065-014-9270-9 25143069 PMC4150993

[B62] Richards M. Deary I. J. (2005). A life course approach to cognitive reserve: A model for cognitive aging and development? *Ann. Neurol.* 58 617–622. 10.1002/ana.20637 16178025

[B63] Richards M. Sacker A. (2003). Lifetime antecedents of cognitive reserve. *J. Clin. Exp. Neuropsychol.* 25 614–624. 10.1076/jcen.25.5.614.14581 12815499

[B64] Rubinov M. Kötter R. Hagmann P. Sporns O. (2009). Brain connectivity toolbox: A collection of complex network measurements and brain connectivity datasets. *NeuroImage* 47:S169. 10.1016/S1053-8119(09)71822-1

[B65] Sadiq M. U. Langella S. Giovanello K. S. Mucha P. J. Dayan E. (2021). Accrual of functional redundancy along the lifespan and its effects on cognition. *NeuroImage* 229:117737. 10.1016/j.neuroimage.2021.117737 33486125 PMC8022200

[B66] Saenz J. L. Downer B. Garcia M. A. Wong R. (2018). Cognition and context: Rural–urban differences in cognitive aging among older mexican adults. *J. Aging Health* 30 965–986. 10.1177/0898264317703560 28553815 PMC5623618

[B67] Saenz J. L. Downer B. Garcia M. A. Wong R. (2022). Rural/urban dwelling across the life-course and late-life cognitive ability in Mexico. *SSM - Popul. Health* 17:101031. 10.1016/j.ssmph.2022.101031 35118187 PMC8800130

[B68] Salat D. H. (2004). Thinning of the cerebral cortex in aging. *Cereb. Cortex* 14 721–730. 10.1093/cercor/bhh032 15054051

[B69] Santabárbara J. Gracía-Rebled A. C. López-Antón R. Tomás C. Lobo E. Marcos G. (2019). The effect of occupation type on risk of Alzheimer’s disease in men and women. *Maturitas* 126 61–68. 10.1016/j.maturitas.2019.05.008 31239120

[B70] Seitz-Holland J. Haas S. S. Penzel N. Reichenberg A. Pasternak O. (2024). BrainAGE, brain health, and mental disorders: A systematic review. *Neurosci. Biobehav. Rev.* 159:105581. 10.1016/j.neubiorev.2024.105581 38354871 PMC11119273

[B71] Solé-Padullés C. Bartrés-Faz D. Junqué C. Vendrell P. Rami L. Clemente I. C. (2009). Brain structure and function related to cognitive reserve variables in normal aging, mild cognitive impairment and Alzheimer’s disease. *Neurobiol. Aging* 30 1114–1124. 10.1016/j.neurobiolaging.2007.10.008 18053618

[B72] Speer M. E. Soldan A. (2014). Cognitive reserve modulates ERPs associated with verbal working memory in healthy younger and older adults. *Neurobiol. Aging* 36:1424. 10.1016/j.neurobiolaging.2014.12.025 25619663 PMC4346428

[B73] Spreng R. N. Setton R. Alter U. Cassidy B. N. Darboh B. DuPre E. (2022). Neurocognitive aging data release with behavioral, structural and multi-echo functional MRI measures. *Sci. Data* 9:119. 10.1038/s41597-022-01231-7 35351925 PMC8964687

[B74] Staff R. T. Murray A. D. Deary I. J. Whalley L. J. (2004). What provides cerebral reserve? *Brain J. Neurol.* 127 1191–1199. 10.1093/brain/awh144 15047587

[B75] Steffener J. (2021). Education and age-related differences in cortical thickness and volume across the lifespan. *Neurobiol. Aging* 102 102–110. 10.1016/j.neurobiolaging.2020.10.034 33765423 PMC8126642

[B76] Steffener J. Brickman A. M. Rakitin B. C. Gazes Y. Stern Y. (2009). The impact of age-related changes on working memory functional activity. *Brain Imaging Behav.* 3 142–153. 10.1007/s11682-008-9056-x 19536354 PMC2697064

[B77] Steffener J. Reuben A. Rakitin B. C. Stern Y. (2011). Supporting performance in the face of age-related neural changes: Testing mechanistic roles of cognitive reserve. *Brain Imaging Behav.* 5 212–221. 10.1007/s11682-011-9125-4 21607547 PMC3169844

[B78] Steffener J. Stern Y. (2012). Exploring the neural basis of cognitive reserve in aging. *Biochim. Biophys. Acta* 1822 467–473. 10.1016/j.bbadis.2011.09.012 21982946 PMC3264833

[B79] Steinberg N. Parisi J. M. Feger D. M. Clay O. J. Willis S. L. Ball K. K. (2023). Rural-urban differences in cognition: Findings from the advanced cognitive training for independent and vital elderly trial. *J. Aging Health* 35 107S–118S. 10.1177/08982643221102718 35604034

[B80] Stern Y. (2002). What is cognitive reserve? Theory and research application of the reserve concept. *J. Int. Neuropsychol. Soc. JINS* 8 448–460. 11939702

[B81] Stern Y. (2009). Cognitive reserve. *Neuropsychologia* 47 2015–2028. 10.1016/j.neuropsychologia.2009.03.004 19467352 PMC2739591

[B82] Stern Y. Albert M. Barnes C. A. Cabeza R. Pascual-Leone A. Rapp P. R. (2023). A framework for concepts of reserve and resilience in aging. *Neurobiol. Aging* 124 100–103. 10.1016/j.neurobiolaging.2022.10.015 36653245 PMC10424718

[B83] Stern Y. Barnes C. A. Grady C. Jones R. N. Raz N. (2019). Brain reserve, cognitive reserve, compensation, and maintenance: Operationalization, validity, and mechanisms of cognitive resilience. *Neurobiol. Aging* 83 124–129. 10.1016/j.neurobiolaging.2019.03.022 31732015 PMC6859943

[B84] Stern Y. Gazes Y. Razlighi Q. Steffener J. Habeck C. (2018). A task-invariant cognitive reserve network. *NeuroImage* 178 36–45. 10.1016/j.neuroimage.2018.05.033 29772378 PMC6409097

[B85] Stern Y. Gurland B. Tatemichi T. K. Tang M. X. Wilder D. Mayeux R. (1994). Influence of education and occupation on the incidence of Alzheimer’s disease. *JAMA* 271 1004–1010. 8139057

[B86] Stern Y. Habeck C. Moeller J. Scarmeas N. Anderson K. E. Hilton H. J. (2005). Brain networks associated with cognitive reserve in healthy young and old adults. *Cereb. Cortex* 15 394–402. 10.1093/cercor/bhh142 15749983 PMC3025536

[B87] Stern Y. Zarahn E. Habeck C. Holtzer R. Rakitin B. C. Kumar A. (2007). A common neural network for cognitive reserve in verbal and object working memory in young but not old. *Cereb. Cortex N. Y. N .* 18:959. 10.1093/cercor/bhm134 17675368 PMC2519015

[B88] Storsve A. B. Fjell A. M. Tamnes C. K. Westlye L. T. Overbye K. Aasland H. W. (2014). Differential longitudinal changes in cortical thickness, surface area and volume across the adult life span: Regions of accelerating and decelerating change. *J. Neurosci.* 34 8488–8498. 10.1523/JNEUROSCI.0391-14.2014 24948804 PMC6608217

[B89] Subramaniapillai S. Almey A. Natasha Rajah M. Einstein G. (2021). Sex and gender differences in cognitive and brain reserve: Implications for Alzheimer’s disease in women. *Front. Neuroendocrinol.* 60:100879. 10.1016/j.yfrne.2020.100879 33137359

[B90] Sundermann E. E. Maki P. M. Rubin L. H. Lipton R. B. Landau S. Biegon A. (2016). Female advantage in verbal memory. *Neurology* 87 1916–1924. 10.1212/WNL.0000000000003288 27708128 PMC5100712

[B91] Tromp D. Dufour A. Lithfous S. Pebayle T. Després O. (2015). Episodic memory in normal aging and Alzheimer disease: Insights from imaging and behavioral studies. *Ageing Res. Rev.* 24 232–262. 10.1016/j.arr.2015.08.006 26318058

[B92] Valenzuela M. J. Sachdev P. (2006). Brain reserve and dementia: A systematic review. *Psychol. Med.* 36 441–454. 10.1017/S0033291705006264 16207391

[B93] Varangis E. Habeck C. G. Razlighi Q. R. Stern Y. (2019). The effect of aging on resting state connectivity of predefined networks in the brain. *Front. Aging Neurosci.* 11:234. 10.3389/fnagi.2019.00234 31555124 PMC6737010

[B94] Zarahn E. Rakitin B. Abela D. Flynn J. Stern Y. (2007). Age-related changes in brain activation during a delayed item recognition task. *Neurobiol. Aging* 28 784–798. 10.1016/j.neurobiolaging.2006.03.002 16621168

[B95] Zhang B. Zhang S. Feng J. Zhang S. (2023). Age-level bias correction in brain age prediction. *NeuroImage Clin.* 37:103319. 10.1016/j.nicl.2023.103319 36634514 PMC9860514

