# Supplementary material for: Investigating dynamic brain functional redundancy as a mechanism of cognitive reserve
Source: Front Aging Neurosci. 2025 Feb 4;17:1535657. doi: 10.3389/fnagi.2025.1535657 (PMC11832541; doi:10.3389/fnagi.2025.1535657)
Supplement: Supplementary file 1 [file Data_Sheet_1.DOCX]

Supplementary Material

# Supplementary Tables

|  | **PC1** | **PC2** | **PC3** |
| --- | --- | --- | --- |
| **PC1** | **0.96** | -0.07 | 0.22 |
| **PC2** | -0.11 | **0.64** | 0.65 |
| **PC3** | -0.20 | -0.43 | **0.65** |

**Supplementary Table 1.** Tucker’s congruency coefficients for factor loading matrices of young and old subgroups.

| Cognitive test | **PC1 (old)** | **PC1 (young)** | **PC2 (old)** | **PC2 (young)** | **PC3 (old)** | **PC3 (young)** |
| --- | --- | --- | --- | --- | --- | --- |
| Verbal Paired Associates: Immediate Recall | 0.44 | 0.37 | 0.00 | -0.29 | -0.22 | -0.15 |
| Verbal Paired Associates: Delayed Recall | 0.43 | 0.35 | -0.01 | -0.31 | -0.21 | -0.10 |
| Verbal Paired Associates: Delayed Free Recall | 0.40 | 0.29 | -0.07 | -0.13 | -0.11 | -0.14 |
| Associative Recall | 0.42 | 0.41 | -0.07 | -0.07 | -0.08 | -0.02 |
| NIH Cognition Auditory Verbal Learning | 0.29 | 0.34 | -0.31 | -0.15 | 0.00 | -0.06 |
| NIH Cognition Picture Sequence Memory | 0.25 | 0.29 | -0.20 | -0.14 | 0.09 | -0.12 |
| Shipley Vocabulary | 0.23 | 0.29 | 0.45 | 0.42 | 0.31 | 0.08 |
| NIH Cognition Picture Vocabulary | 0.18 | 0.27 | 0.45 | 0.45 | 0.31 | 0.05 |
| NIH Cognition Oral Reading Recognition | 0.17 | 0.15 | 0.26 | 0.48 | 0.50 | 0.03 |
| Trail Making Task | -0.07 | -0.06 | 0.05 | -0.27 | -0.14 | 0.24 |
| NIH Cognition Flanker | -0.03 | 0.11 | -0.41 | -0.09 | 0.46 | 0.68 |
| NIH Cognition Dimensional Change Card Sort | 0.01 | 0.11 | -0.43 | -0.12 | 0.39 | 0.63 |
| NIH Cognition List Sort Working Memory | 0.14 | 0.27 | -0.18 | 0.23 | 0.25 | 0.08 |

**Supplementary Table 2.** Principal component coefficients of each cognitive test on the first three principal components for young and old subgroups separately.

| Predictor | **PC1: Episodic** | | | | **PC2: Semantic** | | | | **PC3: Executive** | | | | |
| --- | --- | --- | --- | --- | --- | --- | --- | --- | --- | --- | --- | --- | --- |
|  | β | SE | t | p | β | SE | t | p | β | SE | t | p |  |
| Cortical thickness | 2.253 | 1.155 | 1.950 | 0.052 | **3.616** | **1.243** | **2.908** | **0.004** | -0.010 | 0.916 | -0.011 | 0.992 |  |
| BFR | -0.006 | 0.006 | -0.976 | 0.330 | 0.004 | 0.006 | 0.641 | 0.522 | **0.011** | **0.005** | **2.371** | **0.019** |  |
| Cortical thickness*BFR | -0.031 | 0.048 | -0.641 | 0.522 | -0.037 | 0.052 | -0.717 | 0.474 | 0.022 | 0.038 | 0.582 | 0.561 |  |
| Site | -0.144 | 0.215 | -0.672 | 0.502 | **-1.148** | **0.231** | **-4.963** | **<.001** | **0.740** | **0.170** | **4.343** | **<.001** |  |
| Sex | **0.854** | **0.159** | **5.359** | **<.001** | -0.040 | 0.172 | -0.232 | 0.817 | **-0.418** | **0.126** | **-3.305** | **0.001** |  |
| Age | **-0.075** | **0.005** | **-14.61** | **<.001** | **0.028** | **0.005** | **5.020** | **<.001** | -0.008 | 0.004 | -1.952 | 0.052 |  |
| Fit  Res. SE  R^2^  Adj. R^2^  F  p | **1.27**  **0.71**  **0.70**  **104**  **<.001** | | | | **1.36**  **0.15**  **0.13**  **7.253**  **<.001** | | | | **1.00**  **0.16**  **0.14**  **7.971**  **<.001** | | | | |

**Supplementary Table 3.** Results of multivariate linear regression model across subjects with cortical thickness as age-related brain change controlled for chronological age.

| Predictor | **PC1: Episodic** | | | | **PC2: Semantic** | | | | **PC3: Executive** | | | |
| --- | --- | --- | --- | --- | --- | --- | --- | --- | --- | --- | --- | --- |
|  | β | SE | t | p | β | SE | t | p | β | SE | t | p |
| GM volume | -1.128 | 5.354 | -0.211 | 0.833 | 11.109 | 5.772 | 1.925 | 0.055 | -0.425 | 4.218 | -0.101 | 0.920 |
| BFR | -0.003 | 0.006 | -0.506 | 0.613 | 0.006 | 0.007 | 0.973 | 0.332 | **0.010** | **0.005** | **2.065** | **0.040** |
| GM volume*BFR | 0.061 | 0.240 | 0.253 | 0.801 | 0.080 | 0.259 | 0.308 | 0.758 | -0.044 | 0.189 | -0.230 | 0.818 |
| Site | -0.079 | 0.214 | -0.366 | 0.715 | **-1.071** | **0.231** | **-4.632** | **<.001** | **0.741** | **0.170** | **4.387** | **<.001** |
| Sex | **0.840** | **0.163** | **5.152** | **<.001** | -0.139 | 0.176 | -0.789 | 0.431 | **-0.421** | **0.128** | **-3.277** | **0.001** |
| Age | **-0.083** | **0.006** | **-14.72** | **<.001** | **0.025** | **0.006** | **4.132** | **<.001** | -0.008 | 0.004 | -1.859 | 0.064 |
| Fit  Res. SE  R^2^  Adj. R^2^  F  p | **1.27**  **0.71**  **0.70**  **101.9**  **<.001** | | | | **1.37**  **0.13**  **0.11**  **6.404**  **<.001** | | | | **1.00**  **0.16**  **0.14**  **7.914**  **<.001** | | | |

**Supplementary Table 4.** Results of multivariate linear regression model across subjects with GM volume as age-related brain change controlled for chronological age.

| Predictor | **PC1: Episodic** | | | | | **PC2: Semantic** | | | | | **PC3: Executive** | | | | |
| --- | --- | --- | --- | --- | --- | --- | --- | --- | --- | --- | --- | --- | --- | --- | --- |
|  | β | SE | t | p | β | | SE | t | p | β | | SE | t | p |  |
| Cortical thickness | **14.351** | **1.106** | **12.979** | **<.001** | -0.845 | | 0.909 | -0.929 | 0.354 | **1.335** | | **0.640** | **2.087** | **0.038** |  |
| BFR | **-0.012** | **0.006** | **-2.033** | **0.043** | 0.006 | | 0.005 | 1.279 | 0.202 | **0.008** | | **0.004** | **2.278** | **0.024** |  |
| Cortical thickness*BFR | -0.111 | 0.064 | -1.731 | 0.085 | -0.010 | | 0.053 | -0.192 | 0.848 | -0.002 | | 0.037 | -0.046 | 0.964 |  |
| Site | **-0.978** | **0.277** | **-3.535** | **<.001** | **-0.830** | | **0.228** | **-3.647** | **<.001** | **0.725** | | **0.160** | **4.527** | **<.001** |  |
| Sex | **1.034** | **0.219** | **4.712** | **<.001** | -0.111 | | 0.180 | -0.615 | 0.539 | **-0.420** | | **0.127** | **-3.309** | **0.001** |  |
| Fit  Res. SE  R^2^  Adj. R^2^  F  p | **1.74**  **0.45**  **0.44**  **42.02**  **<.001** | | | | | **1.43**  **0.06**  **0.04**  **2.996**  **0.01** | | | | | **1.01**  **0.15**  **0.13**  **9.027**  **<.001** | | | | |

**Supplementary Table 5**. Results of multivariate linear regression model across subjects with cortical thickness as age-related brain change with a longer sliding window of 100 volumes for the creation of dynamic functional connectivity matrices on which the BFR metric was calculated.

| Predictor | **PC1: Episodic** | | | | **PC2: Semantic** | | | | **PC3: Executive** | | | |
| --- | --- | --- | --- | --- | --- | --- | --- | --- | --- | --- | --- | --- |
|  | β | SE | t | p | β | SE | t | p | β | SE | t | p |
| GM volume | **60.444** | **4.630** | **13.053** | **<.001** | **-7.739** | **3.791** | **-2.041** | **0.042** | **5.363** | **2.684** | **1.998** | **0.047** |
| BFR | **-0.016** | **0.006** | **-2.636** | **0.010** | 0.007 | 0.005 | 1.344 | 0.180 | 0.007 | 0.004 | 2.023 | 0.044 |
| GM volume*BFR | -0.101 | 0.307 | -0.328 | 0.743 | 0.063 | 0.251 | 0.252 | 0.801 | -0.140 | 0.178 | -0.786 | 0.433 |
| Site | **-0.602** | **0.279** | **-2.161** | **0.032** | **-0.864** | **0.228** | **-3.787** | **<.001** | **0.741** | **0.162** | **4.587** | **<.001** |
| Sex | **0.545** | **0.221** | **2.470** | **0.014** | -0.051 | 0.181 | -0.280 | 0.780 | **-0.470** | **0.128** | **-3.673** | **<.001** |
| Fit  Res. SE  R^2^  Adj. R^2^  F  p | **1.74**  **0.45**  **0.44**  **42.35**  **<.001** | | | | **1.42**  **0.07**  **0.05**  **3.664**  **0.003** | | | | **1.01**  **0.15**  **0.13**  **9.005**  **<.001** | | | |

**Supplementary Table 6.** Results of multivariate linear regression model across subjects with GM volume as age-related brain change with a longer sliding window of 100 volumes for the creation of dynamic functional connectivity matrices on which the BFR metric was calculated.

| Predictor | **PC1: Episodic** | | | | **PC2: Semantic** | | | | **PC3: Executive** | | | | |
| --- | --- | --- | --- | --- | --- | --- | --- | --- | --- | --- | --- | --- | --- |
|  | β | SE | t | p | β | SE | t | p | β | SE | t | p |  |
| Brain age | -0.024 | 0.016 | -1.443 | 0.150 | -0.022 | 0.018 | -1.255 | 0.211 | -0.006 | 0.013 | -0.438 | 0.661 |  |
| BFR | -0.004 | 0.005 | -0.943 | 0.347 | 0.003 | 0.005 | 0.557 | 0.578 | **0.008** | **0.004** | **2.251** | **0.025** |  |
| Brain age*BFR | <.001 | <.001 | -0.447 | 0.655 | <.001 | <.001 | 0.805 | 0.422 | <.001 | <.001 | 1.425 | 0.155 |  |
| Site | -0.036 | 0.212 | -0.167 | 0.867 | **-0.989** | **0.231** | **-4.287** | **<.001** | **0.766** | **0.167** | **4.590** | **<.001** |  |
| Sex | **0.826** | **0.160** | **5.156** | **<.001** | -0.105 | 0.174 | -0.601 | 0.548 | **-0.451** | **0.126** | **-3.577** | **<.001** |  |
| Age | **-0.060** | **0.016** | **-3.767** | **<.001** | **0.037** | **0.017** | **2.178** | **0.030** | -0.002 | 0.012 | -0.190 | 0.849 |  |
| Fit  Res. SE  R^2^  Adj. R^2^  F  p | **1.27**  **0.71**  **0.70**  **103.2**  **<.001** | | | | **1.38**  **0.12**  **0.10**  **6.006**  **<.001** | | | | **1.00**  **0.17**  **0.15**  **8.518**  **<.001** | | | | |

**Supplementary Table 7.** Results of multivariate linear regression model across subjects with brain age as age-related brain change controlled for chronological age with a longer sliding window of 100 volumes for the creation of dynamic functional connectivity matrices on which the BFR metric was calculated.

| Predictor | **Episodic Score** | | | | **Semantic Score** | | | | | **Executive Score** | | | |
| --- | --- | --- | --- | --- | --- | --- | --- | --- | --- | --- | --- | --- | --- |
|  | β | SE | t | p | β | SE | t | p | β | | SE | t | p |
| Cortical thickness | **256.0** | **29.65** | **8.633** | **<.001** | **-42.27** | **6.837** | **-6.183** | **<.001** | **54.12** | | **7.335** | **7.379** | **<.001** |
| BFR | **-0.694** | **0.209** | **-3.325** | **0.001** | **0.166** | **0.048** | **3.440** | **<.001** | -0.014 | | 0.052 | -0.270 | 0.787 |
| Cortical thickness*BFR | **-5.085** | **1.754** | **-2.898** | **0.004** | 0.418 | 0.405 | 1.034 | 0.302 | -0.398 | | 0.434 | -0.917 | 0.360 |
| Site | **-30.34** | **7.789** | **-3.895** | **<.001** | **-6.590** | **1.796** | **-3.668** | **<.001** | **5.447** | | **1.927** | **2.827** | **0.005** |
| Sex | **21.00** | **5.870** | **3.578** | **<.001** | **-3.495** | **1.354** | **-2.581** | **0.010** | -0.335 | | 1.452 | -0.230 | 0.818 |
| Fit  Res. SE  R^2^  Adj. R^2^  F  p | **46.7**  **0.33**  **0.32**  **25.26**  **<.001** | | | | **10.77**  **0.20**  **0.19**  **12.76**  **<.001** | | | | | **11.55**  **0.20**  **0.19**  **12.81**  **<.001** | | | |

**Supplementary Table 8**. Results of multivariate linear regression model across subjects with cortical thickness as age-related brain change with the outcome variables as individual cognitive test scores. For the episodic domain, we used the Associative Recall test scores as outcome; for the semantic domain, we used the NIH Cognition Picture Vocabulary test scores and for the executive domain we used the NIH Cognition Flanker test scores as outcome.

| Predictor | **Episodic Score** | | | | **Semantic Score** | | | | **Executive Score** | | | | |
| --- | --- | --- | --- | --- | --- | --- | --- | --- | --- | --- | --- | --- | --- |
|  | β | SE | t | p | β | SE | t | p | β | SE | t | p |  |
| GM volume | **1070** | **125.3** | **8.540** | **<.001** | **-200.1** | **28.24** | **-7.086** | **<.001** | **223.0** | **31.03** | **7.187** | **<.001** |  |
| BFR | **-0.644** | **0.221** | **-2.920** | **0.004** | **0.156** | **0.050** | **3.140** | **0.002** | -0.019 | 0.055 | -0.356 | 0.722 |  |
| GM volume*BFR | -13.16 | 8.779 | -1.499 | 0.135 | 1.256 | 1.978 | 0.635 | 0.526 | -2.552 | 2.174 | -1.174 | 0.241 |  |
| Site | **-25.48** | **7.824** | **-3.257** | **0.001** | **-7.503** | **1.763** | **-4.255** | **<.001** | **6.485** | **1.937** | **3.347** | **<.001** |  |
| Sex | **12.19** | **5.936** | **2.053** | **0.041** | -1.842 | 1.338 | -1.377 | 0.170 | -2.297 | 1.470 | -1.563 | 0.119 |  |
| Fit  Res. SE  R^2^  Adj. R^2^  F  p | **46.84**  **0.33**  **0.32**  **24.83**  **<.001** | | | | **10.55**  **0.23**  **0.22**  **15.38**  **<.001** | | | | **11.6**  **0.20**  **0.18**  **12.34**  **<.001** | | | | |

**Supplementary Table 9.** Results of multivariate linear regression model across subjects with GM volume as age-related brain change with the outcome variables as individual cognitive test scores. For the episodic domain, we used the Associative Recall test scores as outcome; for the semantic domain, we used the NIH Cognition Picture Vocabulary test scores and for the executive domain we used the NIH Cognition Flanker test scores as outcome.

| Predictor | **Episodic Score** | | | | **Semantic Score** | | | | **Executive Score** | | | | |
| --- | --- | --- | --- | --- | --- | --- | --- | --- | --- | --- | --- | --- | --- |
|  | β | SE | t | p | β | SE | t | p | β | SE | t | p |  |
| Brain age | **-1.619** | **0.549** | **-2.948** | **0.003** | -0.089 | 0.119 | -0.746 | 0.457 | -0.026 | 0.014 | -0.190 | 0.849 |  |
| BFR | **-0.461** | **0.222** | **-2.081** | **0.038** | 0.050 | 0.048 | 1.042 | 0.298 | 0.064 | 0.059 | 1.150 | 0.251 |  |
| Brain age*BFR | 0.010 | 0.009 | 1.129 | 0.260 | 0.002 | 0.002 | 1.009 | 0.314 | <.001 | 0.002 | 0.010 | 0.992 |  |
| Site | **-15.96** | **7.283** | **-2.192** | **0.029** | **-8.930** | **1.583** | **-5.640** | **<.001** | **8.008** | **1.835** | **4.364** | **<.001** |  |
| Sex | **16.84** | **5.359** | **3.142** | **0.002** | **-2.994** | **1.165** | **-2.569** | **0.011** | -1.079 | 1.350 | -0.799 | 0.425 |  |
| Age | 0.154 | 0.532 | 0.289 | 0.773 | **0.394** | **0.116** | **3.410** | **<.001** | **-0.284** | **0.134** | **-2.118** | **0.035** |  |
| Fit  Res. SE  R^2^  Adj. R^2^  F  p | **42.58**  **0.45**  **0.43**  **34.08**  **<.001** | | | | **9.26**  **0.41**  **0.40**  **29.5**  **<.001** | | | | **10.73**  **0.31**  **0.30**  **19.31**  **<.001** | | | | |

**Supplementary Table 10.** Results of multivariate linear regression model across subjects with brain age as age-related brain change controlled for chronological age with the outcome variables as individual cognitive test scores. For the episodic domain, we used the Associative Recall test scores as outcome; for the semantic domain, we used the NIH Cognition Picture Vocabulary test scores and for the executive domain we used the NIH Cognition Flanker test scores as outcome.
